# Single injection technique with ultrasound-guided superficial cervical fascia block combined with brachial plexus block in clavicular surgery: a prospective randomized comparative trial

**DOI:** 10.1186/s12871-023-02333-4

**Published:** 2023-11-07

**Authors:** Anneng Zhou, Ying Wang, Yonghong Cheng, Mei He, Yongting Duan, Dongfang Qin, Mengbi Jiang

**Affiliations:** 1 Department of Anesthesiology, Chongqing Wansheng Economic and Technological Development Zone People’s Hospital, No. 43 Wandong North Road, Chongqing, People’s Republic of China; 2 Department of Orthopaedics, Chongqing Wansheng Economic and Technological Development Zone People’s Hospital, Chongqing, People’s Republic of China; 3 Institute of Anesthesiology and Critical Care Medicine, Three Gorges University & Yichang Central People’s Hospital, No. 183 Yiling Avenue, Wujiagang District, Yichang, Hubei Province 443003 People’s Republic of China

**Keywords:** Clavicle, Superficial cervical plexus, Superficial cervical fascia, Supraclavicular nerve

## Abstract

**Background:**

To investigate the effects of a single injection technique with ultrasound-guided superficial cervical fascia block combined with brachial plexus block in clavicular surgery.

**Methods:**

Forty patients, 25 males and 15 females, aged 18–85 years with ASA class I or II underwent unilateral clavicular fracture internal fixation. The patients were randomly divided into a superficial cervical plexus block group (group S, n = 20) and a superficial cervical fascia block group (group F, n = 20). First, the brachial plexus of the intermuscular sulcus of all patients was blocked with an ultrasound-guided injection of one injection with 15ml 0.33% ropivacaine 15ml in both groups. Second, the superficial cervical plexus was blocked by another injection of 5-8ml 0.33% ropivacaine in group S, and the superficial cervical fascia was blocked by an injection with 5-8ml 0.33% ropivacaine in Group F. We evaluated operation time, onset time of anaesthesia, effective time and the grades of nerve block effect in the two groups. Additionally, we evaluated the incidences of local anaesthetic poisoning, hoarseness, dyspnoea, and postoperative nausea and vomiting, and the number of patients requiring remedial analgesia within 24 h. Repeated measurements were analysed by repeated data analysis of variance, and count data were compared by the χ2 test. A P value < 0.05 was considered statistically significant.

**Results:**

The operation time and onset time in Group F were significantly shorter than those in group S (P < 0.05); the effect of intraoperative block was better than that in group S (P < 0.05), and the effective time was significantly longer in group F than in group S (P < 0.05). However, no severe case of dyspnoea, local anaesthetic poisoning or hoarseness after anaesthesia occurred in either of two groups. There was no significant difference in the rate of postoperative salvage analgesia or that of postoperative nausea and vomiting between the two groups.

**Conclusions:**

The application of the single injection technique with ultrasound-guided superficial cervical fascia block combined with brachial plexus block in clavicular surgery is beneficial because it shortens the operation time, has a faster onset, produces a more effective block and prolongs the longer analgesia time.

**Trial registration:**

Chinese Clinical Trial Registry**-** ChiCTR2200064642(13/10/2022).

## Background

Nerve block is a commonly used anaesthesia method in the operation of clavicle fractures. Successful nerve block anaesthesia requires simultaneous block of the anterior rami of the spinal nerves (C3-C7) [1]. Because of the superficial and anterior location of the clavicle, complete nerve block often requires blocking the clavicle to clinically derive its sensory innervation from the brachial plexus and cervical plexus clinically [2–8]. Effective block of the supraclavicular nerve in the superficial cervical plexus is the key to ensuring effective block in the clavicular region [9, 10]. The supraclavicular nerve originates from the midpoint of the posterior edge of the sternocleidomastoid muscle, runs in the superficial cervical fascia, and divides into three branches: internal, middle and external, and distributes throughout the lateral part of the neck, the skin of the upper part of the chest wall and the shoulder [11]. It is not clear whether a local anaesthetic injected into the superficial cervical fascia can block the supraclavicular nerve effectively. To draw a conclusion from a clinical perspective, in our trial, we compared the analgesic effect and adverse reactions of ultrasound-guided superficial cervical fascial block combined with intermuscular sulcus brachial plexus block and ultrasound-guided superficial cervical plexus combined with intermuscular sulcus brachial plexus block during the perioperative period of clavicular surgery.

## Methods

**Fig. 1:**
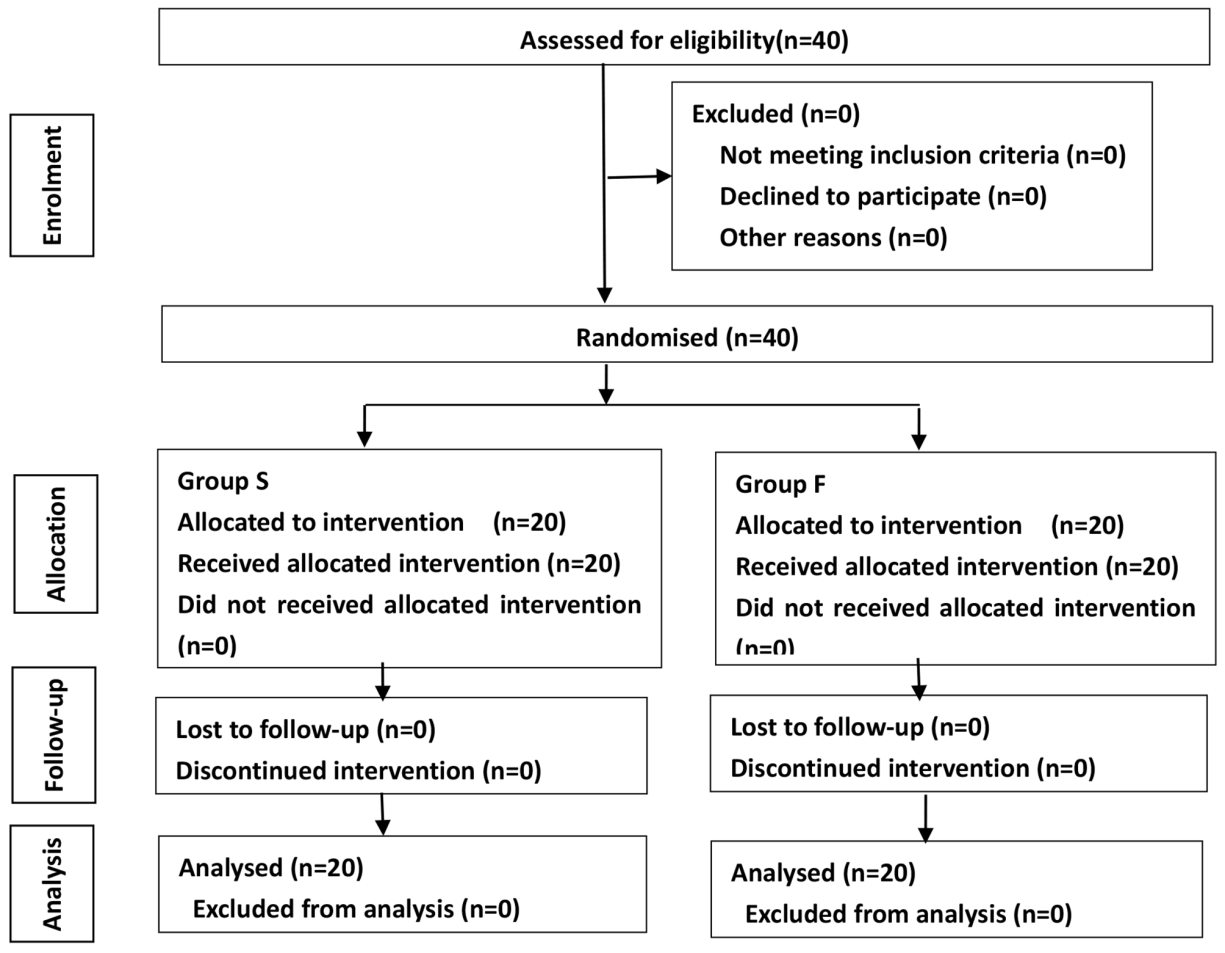
The test process in this trial

The process of this test is as follows (see Fig. 1).

### Recruitment

This trial involved human participants, human material, and human data was therefore conducted in accordance with the Declaration of Helsinki and have been approved by the Ethics Committee of Chongqing Wansheng Economic Development Zone People’s Hospital (2021ky04); the study was prospectively registered at the Chinese Clinical Trial Registry (Number: ChiCTR2200064642, Date: 13/10/2022). All patients were informed of the risks and benefits of participating in this trial and signed a consent form before being enrolled. For this randomized, controlled, single-blind study, 40 patients with unilateral clavicular fracture who were scheduled to undergo open reduction and internal fixation from November 2022 to February 2023 in Chongqing Wansheng Economic Development Zone People’s Hospital were selected. Eligibility criteria for study participation: male or female, aged over 18 years old and an ASA class II to III. Exclusion criteria: concomitant fracture of other parts, serious cardiovascular disease, liver or kidney insufficiency, chronic pain, mental illness, inability to cooperate, language communication disorder, history of allergy to local anaesthetics, puncture site infection, and history of neck surgery, coagulation dysfunction. A nurse who did not participate in the surgery used a random number table to divide the patients into a superficial cervical plexus block group (group S, n = 20) and a superficial cervical fascial block group (group F, n = 20). A random number table was used to randomize the patients to the groups at a 1:1 ratio.

### Ultrasound-guided nerve block

**Fig. 2:**
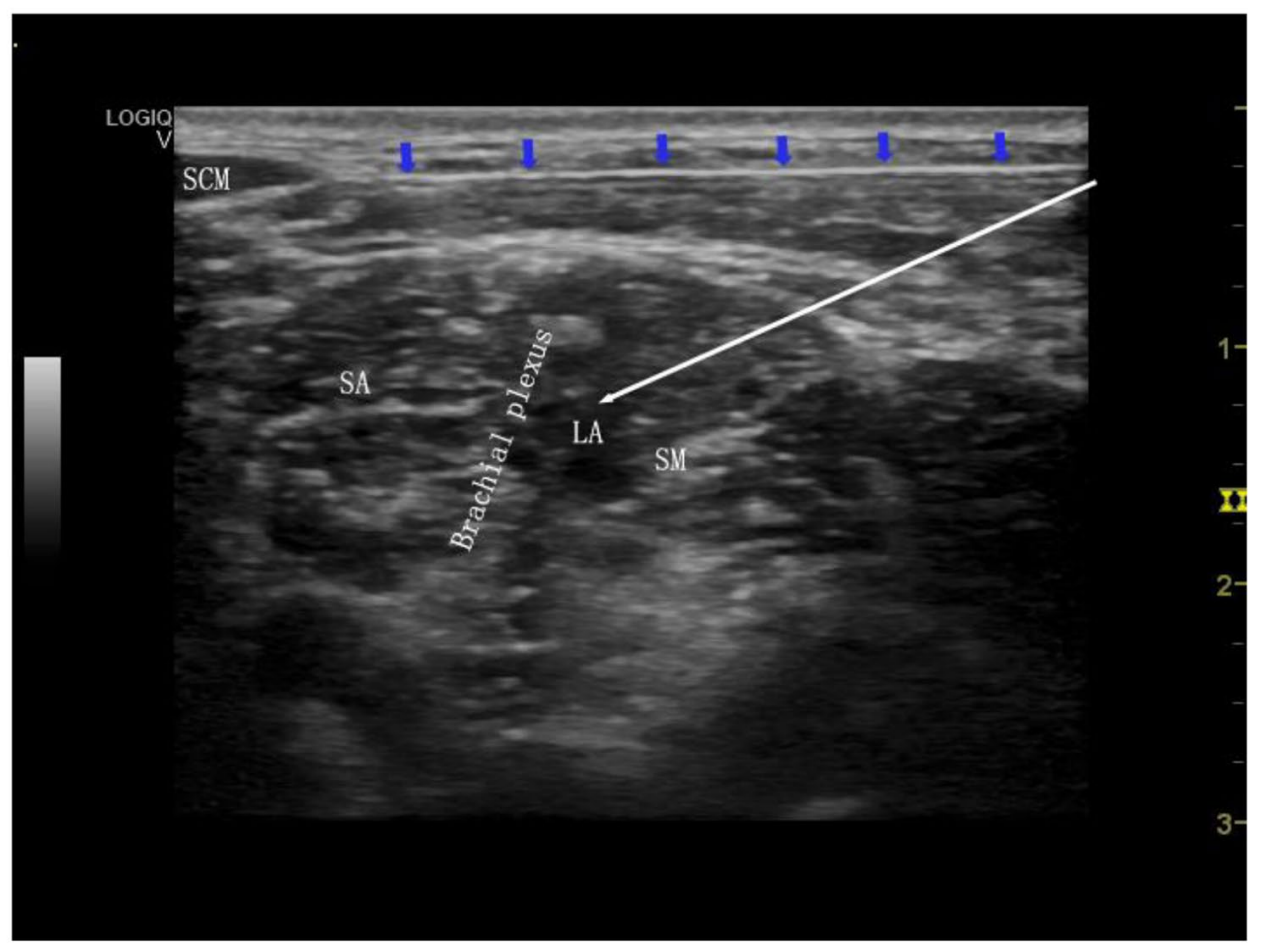
Single injection for superficial cervical fascia block combined with brachial plexus block under ultrasound guidance. SCM: Chest clavicle muscle, SA: Anterior oblique muscle; SM: Middle oblique muscle; LA: Local anaesthetic; Blue arrow: puncture needle with shallow fascia block of the neck; White arrow: needle route of brachial plexus nerve block

The patient’s head is slightly tilted to the opposite side, a thin pillow is placed under the shoulder, and the high-frequency probe of the ultrasound (6–13 MHz, GE LOGIQ V3, Wuxi, Jiangsu, China) that is routinely maintained on a daily basis is placed parallel to the supraclavicular groove. The subclavian artery and the supraclavicular brachial plexus nerve are found and moved slowly to the head end until the brachial plexus nerve root in the intramuscular groove is observed. The puncture needle is inserted from the outside to the inside of the external jugular vein by in-plane technology. When the tip of the needle reached the periphery of the nerve root, 15 ml 0.33% ropivacaine (Guangdong Jiabo Pharmaceutical Co., Ltd., National Pharmaceutical Approval No. H20133178, Product Batch No. 7-B221103-1) 15ml was injected after no blood was drawn back is injected. In all patients the brachial plexus in the intramuscular groove was first blocked on the same side treated by the clavicular operation using one injection under the guidance of ultrasound. In group S, after the completion of the brachial plexus block in the intermuscular groove by one injection, an ultrasonic probe was directed to the head side of the sternocleidomastoid muscle to find the lateral intersection of the external jugular vein and the sternocleidomastoid muscle. The superficial cervical plexus ran between the superficial cervical fascia and the superficial layer of the deep cervical fascia. The puncture needle was inserted from the outside to the inside in the plane of the puncture needle. After the puncture needle reached the target position, 8 ml of 0.33% ropivacaine was given after no blood was drawn back from the other injection. In group F the ultrasonic probe was not moved after completing the intermuscular groove brachial plexus block, the needle was withdrawn to the distance between the superficial cervical fascia and the superficial layer of the deep cervical fascia, and 8 ml of 0.33% ropivacaine was given to expand the fascia of the same injection after confirming that the needle tip was at the target position and no blood was drawn back. The local anaesthetic was diffused along the fascia and injected approximately 4 cm in the ultrasound plane to the front of the sternocleidomastoid muscle (see Fig. 2).

### Operative procedure and block assessment

After entering the room, the patients’ NIBP, ECG, SPO_2_ were monitored, oxygen was inhaled via mask, and a peripheral venous access was established. After intravenous infusion of dexmedetomidine(Yangzijiang Pharmaceutical Group Co., Ltd., National Pharmaceutical Approval No. H20183219, Product Batch No. 22,090,551) 0.6 µg/kg/h, the Ramsay sedation score was maintained at 2–4 points, and the opening was sutured following the infusion of dexmedetomidine. In both groups, anaesthetics were administered by experienced anaesthesiologists skilled in ultrasound-guided nerve block technology and rigorously trained before the trial. The follow-up was completed by anaesthesiologists who were blinded to the grouping. After anaesthesia was completed, the degree of skin pain within the area of the left and right test incisions was compared with the acupuncture method every minute. When there was no pain in the skin on the affected side, pain was no longer assessed, and the operation started. Standard rating of nerve block effect: Grade 1, the block scope is perfect and the patient is painless and quiet; Grade 2, the block scope is not perfect and the patient has pain-free and discomfort, the operation is completed after an intravenous injection a small amount of opioid drugs; Grade 3, the block scope is not perfect and the patient has obvious pain after an intravenous injection of a small amount of opioid drugs, and it is necessary to use local anaesthetics to finish the operation; Grade 4, anaesthesia failure, general anaesthesia is needed to complete the operation. If the nerve block effect is Grade 2 or Grade 3,auxiliary sufentanil is injected slowly and intermittently, with the total amount not exceeding 0.2 µg/kg. If the patient is still in pain, the operator should use 1% lidocaine for local infiltration anaesthesia, with the total amount of lidocaine not exceeding 100 mg. Intravenous analgesia began when patients felt incision pain after surgery. The formulawas as follows: sufentanil 2 µg/kg + flurbiprofen 150 mg was 100 ml, loading dose was 3 ml, maintenance dose was 2 ml/h, PCA dose was 0.5 ml/time, and locking time was 15 min. When the patient’s VAS score was greater than or equal to 4, remedy analgesia was started.

### Observation index

The primary outcomes including block operation time (from the time when the ultrasonic probe contacts the neck skin to the time when the local anaesthetic injection is completed) in minutes, onset time of anaesthesia (after the injection is completed, the acupuncture method is used to test the reduction of skin pain within the scope of the surgical incision every minute, and the onset time of anaesthesia is from the time when the injection is completed to the time when the prick pain disappears) in minutes, the nerve block effect as a percentage, and the time of effective nerve block analgesia (the time of starting analgesia after the operation-the time of starting nerve block operation) in hours were recorded for both groups. The secondary outcomes including the number of patients requiring relief analgesia within 24 h were recorded, and the incidences occurrence of dyspnoea, hoarseness, local anaesthetic poisoning, nausea and vomiting in the two groups were recorded. Additionally, HR, SBP and DBP at the 5 min (T1)after entering the room, at the time of anaesthesia induction (T2), at 5 min after skin incision (T3) and at the time of leaving the operating room (T4) were recorded for the two groups.

### Data statistics

The incidence of perfect anaesthesia effect of the two nerve block methods in the preliminary experiment was 60% and 95%. The power for the primary outcome block effect is calculated based on a two-sided t test with a significance level of 5% and 80% power. Therefore, a total sample size of 40 cases was selected based on the sample size calculation formula and referring to the http://powerandsamplesize.comwebsite. All data were analysed by SPSS 17.0 software. Normally distributed measurement data are expressed as the mean ± standard deviation (X ± S); and the independent sample t test was used for comparisons between groups; repeated data analysis of variance was used for repeated measurement data. The counting data were expressed in cases (%) and compared between the groups using χ2 test. A P value < 0.05 was considered statistically significant. For each group, all participants were included in each analysis and the analysis was of the original assigned groups.

## Results

From November 2022 to February 2023, our test was conducted in accordance with the intention-to-treat method. Forty patients were eligible for enrolment and agreed to participate, and no patient was excluded (see Fig. 1). All 40 patients completed the test and were randomly divided between two groups of 20 patients each. There was no significant difference in sex, age, height, weight, BMI, ASA grade, surgical method or duration of surgery between the two groups (P > 0.05) (see Table 1).

**Table 1:** Patient characteristics and clinical data presented as the mean ± standard deviation or percentages as appropriate

Variables	Group S (n = 20)	Group F (n = 20)	p value
Sex (male/female)	55%/45%	70%/30%	0.523
Age (years)	49.4 ± 12.9	54.8 ± 9.7	0.138
Height(cm)	163.5 ± 7.0	164.4 ± 7.8	0.688
Weight(kg)	65.1 ± 12.2	64.6 ± 9.6	0.892
Body Mass Index (kg.m^− 2^)	24.2 ± 3.3	23.9 ± 2.7	0.705
ASA(I/II)	80%/20%	80%/20%	1.000
Surgical method(internal fixation/ removal)	55%/45%	65%/35%	0.748
Duration of surgery (minutes)	102.8 ± 39.0	122.8 ± 44.1	0.138

### Primary outcomes

The block operation time and onset time of group F were significantly shorter than those of group S (P < 0.05); the patients in the two groups underwent the surgery under nerve block, no anaesthesia conversion was needed, and the block effect during the operation was better than that of group S (P < 0.05); and the effective analgesia time was significantly longer than that of group S (P < 0.05)(see Table 2).

**Table 2:** Comparison of neural block operation time, onset time, block effect operation time and effective analgesia time between the two groups

Variables	Group S (n = 20)	Group F (n = 20)	p value
Block operation time(min)	6.34 ± 0.98	5.47 ± 0.96	0.007
Onset time(min)	3.60 ± 1.47	2.60 ± 1.39	0.033
Block effect(1/2/3/4)	60%/30%/10%/0	95%/5%/0/0	0.023
Effective analgesia time (h)	6.30 ± 3.63	10.45 ± 3.14	< 0.001

### Secondary outcomes

None of the patients in either of the two groups had severe dyspnoea, local anaesthetic poisoning reactions or hoarseness after anaesthesia. One patient in group F required remedial analgesia within 24 h, one patient in group F and two patients in group experienced nausea and vomiting. There was a statistically significant difference in heart rate at the time of anaesthesia induction (T2) between the two groups(P < 0.05); but there was no statistically significant difference in vital signs between the two groups at other time points(P > 0.05) (see Table 3).

**Table 3:** Comparison of intraoperative vital signs between the two groups

Variables	Group	T1	T2	T3	T4
HR(bpm)	S(n = 20)	79.2 + 10.8	79.9 + 9.7	77.1 + 10.1	73.9 + 9.3
	F(n = 20)	73.3 + 10.1	73.1 + 9.6^a^	71.3 + 10.9	68.4 + 12.8
SBP(mmHg)	S(n = 20)	126.4 + 18.8	127.6 + 17.3	122.0 + 18.8	119.5 + 16.9
	F(n = 20)	128.7 + 22.3	130.8 + 24.9	121.5 + 15.3	115.8 + 11.5
DBP(mmHg)	S(n = 20)	79.3 + 9.6	78.5 + 10.2	76.4 + 10.0	75.4 + 10.8
	F(n = 20)	81.1 + 15.0	79.2 + 12.6	76.5 + 12.3	72.6 + 9.9

Comparison with group S, ^a^P<0.05

## Discussion

Clavicular fracture is one of the most common clinical fractures, and incision reduction internal fixation surgery is the main treatment method [12]. This test showed that ultrasound-guided superficial cervical fascia block combined with brachial plexus block has a better block effect, shortens the operation and analgesia onset times and has a longer effective analgesia time than brachial plexus block. With respect to its anatomical location, the clavicle belongs to the upper limb bone, which is controlled by the C3 to C7 spinal nerves. Leurcharusmee P [13] found through anatomy that the supraclavicular nerve (C3 ~ 4) innervates the skin and sternoclavicular joint of the entire clavicular region, while the acromioclavicular joint is innervated by the supraclavicular nerve and the lateral thoracic nerve. The subclavian nerve (C5 ~ 6) innervates the caudal and dorsal sides of one-third of the clavicle, and the lateral thoracic nerve (C5 ~ 7) innervates the caudal part of the clavicle. Successful nerve block anaesthesia in clavicular surgery requires simultaneous block of the supraclavicular nerve in the brachial plexus and superficial cervical plexus. The brachial plexus is relatively thick and stable. With the application of ultrasound, the success rate of blocking is high. Whether the supraclavicular nerve can be successfully blocked is the key to ensuring the anaesthesia effect [9, 10].

At present, ultrasound-guided superficial cervical plexus combined with brachial plexus block is the most widely used method in clinical practice [2–8]. Jiang Xin [14] conducted an ultrasound-guided great auricular nerve assisted localization of superficial cervical plexus block study, in which the rate of complete block of the supraclavicular nerve was only 80%, which was believed to related to the anatomical variation of the supraclavicular nerve [15]. In the clinic, some patients have poor analgesic effects and require the operator to administer additional local anaesthesia or even change to general anaesthesia to complete the operation. The two-point puncture method increases pain in skin puncture patients. In 2014, Valdés-Vilches LF [9] reported that ultrasound-guided localization of the supraclavicular nerve in the intramuscular groove 2 ~ 3 cm above the clavicle and selective supraclavicular nerve block with a low volume (1.5 ~ 2 ml) combined with brachial plexus block with a low volume (8 ~ 15 ml) could provide a satisfactory anaesthetic effect for operation of internal fixation of the clavicle. However, the supraclavicular nerve is too small to identify under ultrasound and its branches and routes are variable [16]; therefore, this approach is difficult to promote in clinical practice. This method of ultrasound-guided superficial cervical fascia block combined with brachial plexus block may have a better anaesthesia effect than previous supraclavicular nerve block or combined anterior cervical plexus block and requires the use of fewer anaesthetics. Relevant studies can be further conducted.

In recent years, ultrasound-guided interfascial plane block has been widely used for regional anaesthesia and pain management of the trunk [17, 18]. The supraclavicular nerve originates from the posterior margin of the midpoint of sternocleidomastoid muscle and runs between the superficial layer of deep fascia and superficial cervical fascia. It is divided into three internal and external branches, distributed in the skin of the lateral neck, the upper part of the chest wall and the shoulder [4]. The strengths of our study are as follows. First, the single-point skin puncture is used to complete the nerve block operation; the other is to successfully complete clavicle operation by combining superficial cervical fascia block and brachial plexus nerve block to achieve anaesthesia. In our study, the superficial cervical fascia block group received an intramuscular groove brachial plexus block through an in-plane technique without moving the ultrasonic probe before withdrawing the needle to the superficial cervical fascia layer and expanding the fascia with local anaesthetics. The needle is sent to the front of the sternocleidomastoid muscle along the expanded fascial space, which can ensure that the internal and external branches of the supraclavicular nerve can be fully blocked. Injecting liquid medicine between the two layers of fasciae is conducive to its diffusion throughout the fascia and and there is less overflow of liquid medicine outside the fascia, so more liquid medicine can act on the nerves in the fascia for a longer time. This is the reason the superficial cervical fascial block group in this study had a shorter block operation time and onset time, a better blocking effect and a longer analgesia time. Without a moving ultrasound probe, it is not necessary to identify the anatomical structure under ultrasound again; thus reducing the blocking operation time.In our study, the reduction in heart rate at T2 in the superficial cervical fascial block group may be related to the completion of both the brachial plexus nerve and the superior clavicle nerve block through a puncture point, which better reduces the pain of patients than two skin punctures. No cases of local anaesthetic poisoning, hoarseness or diaphragmatic paralysis was found in this study, which proves that ultrasound-guided nerve block is safe [19]. It may also be related to the low dosage of anaesthetics used for intramuscular sulcus brachial plexus block [20].

The inadequacies of this study are as follows: first, this study did not discuss the optimal concentration and volume of local anaesthetics for superficial cervical fascial block; second, invasive arterial blood pressure monitoring was not performed; third, the ultrasound contrast monitoring of diaphragm movement was not performed, and the observation of dyspnoea was used to assess whether the sensitivity of the phrenic nerve block was poor, especially for patients with good cardiopulmonary function. In addition, the sample size seems insufficient to detect any adverse effects. Ultrasound-guided nerve block methods cause very few adverse reactions. Therefore, such reactions were not considered for this test, which is a shortcoming of this article, and further research is needed in the future. The scheme provided by this experiment shows obvious advantages, and future research requires more sophisticated methods for further in-depth research.

## Conclusions

In summary ultrasound-guided single-point superficial cervical fascial block combined with intramuscular groove brachial plexus block in clavicular surgery has a short blocking operation time, satisfactory effect, fast onset, long analgesia time and no obvious complications. Further research is needed to identify the optimal concentration and volume of local anaesthetics for superficial cervical fascial block and to observe its possible adverse effects in a sufficient sample size. It may improve patient satisfaction and reduce healthcare-related costs. It is worth promoting in clinical practice.

## Data Availability

The datasets used and/or analysed during the current study are available from the corresponding author upon reasonable request.

## Abbreviations

ASA: American Society of Anaesthesiologists score
NIBP: NonInvasive blood pressure
ECG: Electrocardiogram
SPO_2_: Pulse oxygen saturation
PCA: Patient-controlled analgesia
VAS: Visual analogue score
BMI: Body mass index
